# Eleganolone, a Diterpene from the French Marine Alga *Bifurcaria bifurcata* Inhibits Growth of the Human Pathogens *Trypanosoma brucei* and *Plasmodium falciparum*

**DOI:** 10.3390/md11030599

**Published:** 2013-02-26

**Authors:** Jean-Baptiste Gallé, Barthélémy Attioua, Marcel Kaiser, Anne-Marie Rusig, Annelise Lobstein, Catherine Vonthron-Sénécheau

**Affiliations:** 1 UMR 7200 CNRS, Therapeutic Innovation Laboratory, Faculty of Pharmacy, University of Strasbourg, 64701 Illkirch, France; E-Mails: galle@unistra.fr (J.-B.G.); lobstein@unistra.fr (A.L.); 2 Department of Material Structure Sciences and Technology, University of Cocody, 01 BP 582, Abidjan, Ivory Coast; E-Mail: attioua@yahoo.fr; 3 Swiss Tropical and Public Health Institute, 4002 Basel, Switzerland; E-Mail: kmarcel.Kaiser@unibas.ch; 4 University of Basel, Petersplatz 1, 4003 Basel, Switzerland; 5 CNRS INEE-FRE3484 Marine Mollusks Biology and Associated Ecosystems, University of Caen Basse-Normandie, 14032 Caen Cedex, France; E-Mail: anne-marie.rusig@unicaen.fr

**Keywords:** eleganolone, linear diterpene, *Bifurcaria*, *Plasmodium falciparum*, *Trypanosoma*

## Abstract

Organic extracts of 20 species of French seaweed have been screened against *Trypanosoma brucei rhodesiense* trypomastigotes, the parasite responsible for sleeping sickness. These extracts have previously shown potent antiprotozoal activities *in vitro* against *Plasmodium falciparum* and *Leishmania donovani*. The selectivity of the extracts was also evaluated by testing cytotoxicity on a mammalian L6 cell line. The ethyl acetate extract of the brown seaweed, *Bifurcaria bifurcata*, showed strong trypanocidal activity with a mild selectivity index (IC_50_ = 0.53 µg/mL; selectivity index (SI) = 11.6). Bio-guided fractionation led to the isolation of eleganolone, the main diterpenoid isolated from this species. Eleganolone contributes only mildly to the trypanocidal activity of the ethyl acetate extract (IC_50_ = 45.0 µM, SI = 4.0). However, a selective activity against *P. falciparum* erythrocytic stages *in vitro* has been highlighted (IC_50_ = 7.9 µM, SI = 21.6).

## 1. Introduction

Human African trypanosomiasis (sleeping sickness) is a fatal disease, if untreated. With fewer than 12,000 cases reported per year, trypanosomiasis belongs to the most neglected tropical diseases [1]. The causative agents are *Trypanosoma brucei rhodesiense* in East Africa and *T. b. gambiense* inWest and Central Africa. Therapy of sleeping sickness remains a problem. The available drugs are outdated, complicated to administer and can cause severe adverse reactions [2]. Safe and effective drugs are urgently needed.

Marine macrophytes have shown their extensive biological activity [3,4,5], including antiprotozoal [6,7,8,9,10,11], but very little is known about the compounds responsible for these activities.

As a part of our continuous search for new natural antiprotozoal secondary metabolites, 35 polar (hydroalcoholic) and apolar (ethyl acetate) extracts from 20 species of seaweed from the Normandy coast (France), which we previously reported to have other antiprotozoal activities [6], were screened against cultured trypomastigotes of *Trypanosoma brucei rhodesiense*, as well as for cytotoxicity on a mammalian cell line (L6). Herein, we report the bio-guided fractionation of the most active extract and structure elucidation and antiprotozoal activity of its main constituent.

## 2. Results and Discussion

### 2.1. Selected Species

The sampling resulted in the selection of 20 species of seaweed (Table 1). The samples were collected, as described previously [6].

**Table 1:** Marine algal species selected for the study [6].

Species	Family	Collection site	Collection time
**Chlorophyta**			
*Codium tomentosum* Stackhouse	Codiaceae	Cap Lévy (Manche)	June 2007
*Ulva lactuca* (Linnaeus)	Ulvaceae	Luc-sur-Mer (Calvados)	October 2006
*Ulva clathrata* (Roth) C. Agardh	Ulvaceae	Anse St Martin (Manche)	June 2007
**Heterokontophyta**			
*Bifurcaria bifurcata* R. Ross	Sargassaceae	Cap Lévy (Manche)	June 2007
*Dictyopteris polypodioides* (A.P. de Candolle) J.V. Lamouroux	Dictyotaceae	Barneville (Calvados)	October 2007
*Dictyota dichotoma* (Hudson) J.V. Lamouroux	Dictyotaceae	Anse St Martin (Manche)	June 2007
*Fucus serratus* (Linnaeus)	Fucaceae	Luc-sur-mer (Calvados)	November 2005
*Himanthalia elongata* (Linnaeus)	Himanthaliaceae	Cap Lévy (Manche)	June 2006
*Laminaria digitata* (Linnaeus) J.V. Lamouroux	Laminariaceae	Langrunes-sur-Mer (Calvados)	January 2007
*Pelvetia canaliculata* Decaisne & Thuret	Fucaceae	Cap Lévy (Manche)	June 2006
*Sargassum muticum* (Yendo) Fensholt	Sargassaceae	Cap Lévy (Manche)	June 2006
**Rhodophyta**			
*Calliblepharis jubata* (Goodenough & Woodward) Kützing	Cystocloniaceae	Cap Lévy (Manche)	June 2007
*Chondrus crispus* Stackhouse	Gigartinaceae	Cap Lévy (Manche)	June 2007
*Dilsea carnosa* (Schmidel) Kuntze	Dumontiaceae	Langrune-sur-Mer (Calvados)	January 2007
*Gelidium latifolium* Bornet ex Hauck	Gelidiaceae	Cap Lévy (Manche)	June 2006
*Gracilaria gracilis* (Stackhouse) Steentoft, L.M. Irvine & Farnham	Gracilariaceae	Anse St Martin (Manche)	June 2007
*Grateloupia turuturu* Yamada	Halymeniaceae	St Vaast-la-Hougue (Manche)	September 2007
*Halurus flosculosus* (J. Ellis) Maggs & Hommersand	Ceramiaceae	Anse St Martin (Manche)	June 2007
*Mastocarpus stellatus* (Stackhouse) Guiry	Phyllophoraceae	Cap Lévy (Manche)	June 2006
*Palmaria palmata* (Linnaeus) Kuntze	Palmariaceae	Luc-sur-Mer (Calvados)	November 2005

### 2.2. *In Vitro* Trypanocidal Activity of the Selected Species

The trypanocidal activity of the resultant ethyl acetate and hydroalcoholic extracts were evaluated *in vitro* against *Trypanosoma brucei rhodesiense* trypomastigotes (STIB 900 strain). Extracts were first screened at two concentrations (1.6 and 9.7 µg/mL), and parasite growth inhibition was measured. Extracts for which parasite growth inhibition was greater than 50% at the concentration of 9.7 µg/mL were subsequently assayed to determine their IC_50_. Cytotoxicity to primary mammalian L6 cells was also evaluated to determine the selectivity of its activity. Table 2 presents the IC_50_ values and selectivity indexes (SIs) of the active extracts (ratio of cytotoxic to trypanocidal activity). An SI value >10 is generally considered to indicate antiprotozoal activity not due to general cytotoxicity.

**Table 2:** *In vitro* trypanocidal activity of the active extracts against *T. brucei rhodesiense* trypomastigotes (STIB 900 strain). Data shown are means of two independent assays, which varied <±50%. SI: selectivity index; ratio of cytotoxic activity on L6 cells to trypanocidal activity. E: hydroalcoholic extract, A: ethyl acetate extract.

		IC_50_ (µg/mL)		Selectivity index (SI)
		Antitrypanosomal activity	Cytotoxic activity	
Species	Extract	*T. brucei rhodesiense*	L6 cells	
*B. bifurcata*	E	29.7	76.0	2.6
*B. bifurcata*	A	0.5	6.2	12.4
*C. jubata*	A	23.3	71.5	3.1
*C. crispus*	A	13.6	84.3	6.2
*D. dichotoma*	A	5.8	27.8	4.8
*D. carnosa*	A	15.3	74.0	4.8
*G. latifolium*	A	20.5	62.1	3.0
*G. gracilis*	A	21.5	71.3	3.3
*G. turuturu*	A	10.8	71.2	6.6
*H. flosculosus*	A	22.4	58.7	2.6
*H. elongata*	A	30.3	88.3	2.9
*M. stellatus*	A	19.5	69.1	3.5
*P. canaliculata*	A	7.8	86.7	11.1
*S. muticum*	A	5.8	27.8	4.8
Standards				
Melarsoprol		0.004		
Podophyllotoxin			0.007	

As shown previously for antiplasmodial and leishmanicidal activities [6], the active extracts were almost entirely ethyl acetate extracts (37%), while hydroalcoholic extracts were mainly inactive (2.8%).

Four ethyl acetate extracts showed activity under 10 µg/mL: *B. bifurcata*, *D. dichotoma*, *P. canaliculata* and *S. muticum*. *B. Bifurcaria* and *P. canaliculata* extracts showed selectivity indexes >10, suggesting these extracts could be selectively active against *T. brucei rhodesiense*. The remaining extracts were less active, with IC_50_ values ranging from 10.8 to 29.7 µg/mL. Interestingly, the four most active species belong to the Heterokontophyta. Very little research has been carried out on the antiprotozoal potential of brown alga, and there are only few screening papers available on this subject [7,8,9,10]. Only one of them deals with the species studied here [7]. There were good similarities in the trypanocidal effect of Turkish [9], British [7] and French *D. dichotoma*. Also, French *B. bifurcata*, *P. canaliculata* and *S. muticum* show a similar activity profile to British ones, but with activity about twice as great [7].

*B. bifurcata* was the most active species, with an IC_50_ value of 0.5 µg/mL for the ethyl acetate extract. To our knowledge, in addition to our study and that of Spavieri, there is no data available regarding the antiprotozoal properties of extracts derived from this species. In contrast, cytotoxic activities have been described for methanolic and chloroform/methanol extracts and compounds isolated from ether extracts of *B. bifurcata* [12,13,14].

Indeed, phytochemistry of this species has been widely described. *B. bifurcata* is characterized by polyphenols (phlorotannins) [15,16], fucosterol [17], but mainly by linear oxygenated diterpenes [18,19,20,21,22,23,24,25,26], eleganolone being the major one [27]. Several studies have proven the cytotoxic activities of *B. bifurcata* and/or its secondary metabolites. Bifurcane and analogs showed a cytotoxic effect on fertilized sea urchin eggs (ED_50_ = 4–12 µg/mL) [28]. Bifurcadiol exhibited cytotoxicity against cultured tumor cell lines with ED_50_ values of 4 to 10 µg/mL [29]. Other trihydroxylated acyclic diterpenes were proven to be active *in vitro* against the NSCLC-N6 cell line derived from a human non-small-cell bronchopulmonary carcinoma, with IC_50_ values of 9.5 to 12.3 µg/mL [20]. Eleganolone and analogs showed anti-fouling activity [30,31]. However, no study has been performed on the antiprotozoal activities of its metabolites.

As the ethyl acetate extract has shown significant activity against *T. brucei rhodesiense*, we subjected it to further fractionation in order to isolate and characterize the active ingredients.

### 2.3. Bio-Guided Fractionation of the Most Active Extract Obtained from *B. bifurcata*

The ethyl acetate extract of *B. bifurcata* was fractionated through column chromatography on silica gel using a polarity gradient to generate five main fractions. Bio-guided fractionation was followed by the isolation of the main constituent of the most active fraction. Each fraction or compound was evaluated for trypanocidal activity against *T. brucei rhodesiense* trypomastigotes. Fractions and purified compound were also evaluated for antiprotozoal activities against *T. cruzi* amastigotes and *P. falciparum* intraerythrocytic stages and for cytotoxic activity towards L6 mammalian cells to assess their selectivity of activity (Table 3 and Table 4).

**Table 3:** *In vitro* antiprotozoal activity of fractions obtained from the ethyl acetate crude extract of *B. bifurcata.* Data shown are means of two independent assays, which varied <±50%. SI: selectivity index; ratio of cytotoxic activity on L6 cells to antiprotozoal activity measured against ^a^ *T. brucei rhodesiense*, ^b^ *T. cruzi* and ^c^ *P. falciparum*. * Data from [6].

	Antiprotozoal activity			Cytotoxic activity			
	IC_50_ (µg/mL)			IC_50_ (µg/mL)	SI		
	*T. brucei rhodesiense*	*T. cruzi*	*P. falciparum*	L6 cells	SI ^a^	SI ^b^	SI ^c^
Ethyl acetate extract	0.5	4.1 *	>5 *	6.2 *	12.4	1.5 *	>1 *
*Fractions*							
Fraction 1	12.9	32.4	8.5	40.2	3.1	1.2	4.7
Fraction 2	0.5	9.7	3.8	7.4	15.4	0.8	1.9
Fraction 3	4.3	11.7	3.7	16.9	3.9	1.4	4.6
Fraction 4	14.7	25.0	3.0	55.1	3.7	2.2	18.4
Fraction 5	14.1	32.1	4.8	38.7	2.7	1.2	8.0
*Standard drugs*							
Melarsoprol	0.004						
Benznidazole		0.536					
Chloroquine			0.069				
Artemisinin			0.002				
Podophyllotoxin				0.007			

**Table 4:** *In vitro* antiprotozoal activity of eleganolone, the main component of the most active fraction obtained from the crude ethyl acetate extract of *B. bifurcata*. Data shown are means of two independent assays, which varied <±50%. SI: selectivity index; ratio of cytotoxic activity on L6 cells to antiprotozoal activity measured against ^a^ *T. brucei rhodesiense* and ^b^ *P. falciparum*.

	Antiprotozoal activity			Cytotoxic activity		
	IC_50_ in µM (µg/mL)			IC_50_ (µg/mL)	SI	
	*T. brucei rhodesiense*	*T. cruzi*	*P. falciparum*	L6 cells	SI ^a^	SI ^b^
Eleganolone	45 (13.7)	58 (17.7)	7.9 (2.6)	184 (56.1)	4.0	21.6
*Standard drugs*						
Melarsoprol	0.005 (0.004)					
Benznidazole		1.69 (0.536)				
Chloroquine			0.19 (0.069)			
Artemisinin			0.007 (0.002)			
Podophyllotoxin				0.048 (0.007)		

#### 2.3.1. Fractions

The strong trypanocidal activity detected in the crude ethyl acetate extract (IC_50_ = 0.5 µg/mL) was found in fraction 2 (IC_50_ = 0.5 µg/mL), with a little increase in selectivity (SI = 12.4 and 15.4, respectively) (Table 3). Other fractions were less active and less selective towards *T. brucei rhodesiense*.

#### 2.3.2. Pure Compound

**Figure 1:**
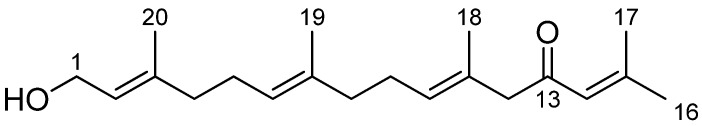
Chemical structure of eleganolone or 2,6,10,14-hexadecatetraen-4-one.

The main constituent of the fraction 2 was isolated by successive column chromatographies on silica gel and identified as eleganolone (Figure 1). Eleganolone was unambiguously identified by comparison of its spectral data with those described in the literature [21], on the basis of chemical and spectral evidence, including ^1^H, ^13^C and Distortionless Enhancement by Polarization Transfer (DEPT) 135 nuclear magnetic resonance (NMR) experiments and High Resolution Mass Spectrometry.

Antiprotozoal activity of eleganolone was then evaluated against the protozoa parasites to check whether or not eleganolone contribute to activity (Table 4).

Eleganolone was less active against *T. brucei rhodesiense* than the fraction from which it has been isolated (IC_50_ = 13.7 µg/mL and 0.5 µg/mL, respectively). Indeed, eleganolone showed only mild trypanocidal activity against *T. brucei rhodesiense*, with a poor selectivity index (SI = 4.0). On the other hand, eleganolone exhibited antiplasmodial activity with a good selectivity (SI = 21.6). The trypanocidal activity, which was lost during the fractionation, could not be attributed to eleganolone, although it is the main component of the active fraction. However, *B. bifurcata* contains a large variety of structurally related oxygenated diterpenoids that could contribute synergistically to the activity of the crude extract. Nevertheless, the fractionation revealed that eleganolone was selectively active against *P. falciparum*, compared to other protozoa or mammalian cells. To the best of our knowledge, antiplasmodial activity has not been reported yet for eleganolone.

## 3. Experimental Section

### 3.1. General Experimental Procedures

Silica gel 60 (230–400 mesh, Merck, Stockholm, Sweden) was used as the stationary phase for column chromatography. ^1^H-NMR and ^13^C-NMR spectra were recorded in deuterated chloroform (CDCl_3_, Eurisotop, Saint Aubin, France) on a Bruker Avance DRX-400 spectrometer at 400 MHz (^1^H) and 100 MHz (^13^C, DEPT 135). The High Resolution Electrospray Ionization Mass Spectroscopy (HREIMS) was performed on an Esquire 3000 plus ion trap mass spectrometer (Bruker Daltonics, Bremen, Germany).

### 3.2. Algae Collection and Identification

The 20 algae species were collected between November 2005 and September 2007 at several locations on the coast of Basse-Normandie. Table 1 reports the collection dates and sites.

Taxonomic determination was performed by Dr. A.-M. Rusig, and voucher specimens of the algae are deposited in the Herbarium of the University of Caen.

### 3.3. Extraction and Compound Isolation

Crude extracts were prepared as described before [6]. Briefly, freeze-dried and ground thallus of *B. bifurcata* (250 g) was extracted at room temperature with ethyl acetate and concentrated to dryness under vacuum. The residue (12 g) was subjected to successive flash and column chromatographies over silica gel (230–400 mesh, Merck) eluting with a cyclohexane-ethyl acetate mixture of increasing polarity to yield five main fractions, labeled 1–5. Separations of fraction 2 (2.5 g) by repeated column chromatography on silica gel eluted with a cyclohexane-ethyl acetate mixture of increasing polarity yielded eleganolone (10.3 mg).

*Eleganolone*: colorless oil; ^1^H NMR (CDCl_3_, 400 MHz) δ 5.36 (1H, t, *J* = 6.8 Hz, H-2), 5.20 (1H, t, *J* = 6.4 Hz, H-10), 5.08 (1H, t, *J* = 6.4 Hz, H-6), 4.11 (1H, d, *J* = 6.8 Hz, H-1), 2.99 (1H, br, s, H-12), 2.04 (2H, t, *J* = 8.0 Hz, H-4, H-8), 1.97 (2H, dt, *J* = 8.0 Hz, 6.4 Hz, H-5, H-9), 1.82 (1H, br, s, H-17), 1.62 (1H, br, s, H-18), 1.55 (3H, br, s, H-16, H-19, H-20); ^13^C NMR (CDCl3, 100 MHz) δ 199.5 (C, C-13), 155.8 (C, C-15), 139.8 (C, C-3), 138.8 (C, C-7), 130.9 (C, C-11), 124.7 (CH, C-14), 123.5 (CH, C-10), 123.0 (CH, C-2), 122.7 (CH, C-6), 59.0 (CH_2_, C-1), 55.3 (CH_2_, C-12), 39.8 (CH_2_, C-4, C-8), 26.7 (CH_2_, C-5), 26.4 (CH_2_, C-9), 25.1 (CH_3_, C-16), 19.9 (CH_3_, C-20), 17.5 (CH_3_ C-17), 17.2 (CH_3_, C-18), 16.9 (CH_3_, C-19); HREIMS [M + Na]^+^ *m/z* 327.23193 (calculated for C_20_H_32_O_2_Na, 327.22945). Spectroscopic data matched those previously published [21].

### 3.4. *In Vitro* Antiprotozoal Assays

The extracts were dissolved in dimethylsulfoxide (DMSO) to obtain a concentration of 10 mg/mL and screened for antiprotozoal activity against *P. falciparum*, *T. cruzi* and *T. brucei rhodesiense* and cytotoxicity against rat skeletal muscle myoblasts (L6 cells). The *in vitro* assays were conducted as described by Scala *et al.* [31]. A brief description is given below.

#### 3.4.1. Activity against *P. falciparum*

*In vitro* activity against erythrocytic stages of *P. falciparum* was determined by a modified [^3^H]-hypoxanthine incorporation assay with the chloroquine- and pyrimethamine-resistant K1 strain [32]. Briefly, parasite cultures incubated in Roswell Park Memorial Institute (RPMI) 1640 medium with 5% Albumax (without hypoxanthine) were exposed to serial drug dilutions in microtiter plates. After 48 h of incubation at 37 °C in a reduced oxygen atmosphere, 0.5 µCi [^3^H]-hypoxanthine was added to each well. Cultures were incubated for a further 24 h before they were harvested onto glass-fiber filters and washed with distilled water. The radioactivity was counted with a Betaplate™ liquid scintillation counter (Wallac, Zurich, Switzerland). The results were recorded as counts per minute (CPM) per well at each drug concentration and expressed as the percentage of untreated controls. IC_50_ values were calculated from graphically plotted dose-response curves by linear interpolation. Chloroquine (Sigma C6628) and artemisinin (Sigma 36,159-3) were used as positive references.

#### 3.4.2. Activity against *Trypanosoma cruzi*

Rat skeletal myoblasts (L6 cells) were seeded in 96-well microtiter plates at 2000 cells/well in 100 μL RPMI 1640 medium with 10% fetal bovine serum (FBS) and 2 mM L-glutamine. After 24 h, the medium was removed and replaced by 100 μL per well containing 5000 trypomastigote forms of *T. cruzi* Tulahuen strain C2C4 with the β-galactosidase (Lac Z) gene [33]. After 48 h, the medium was removed from the wells and replaced by 100 μL fresh medium with or without a serial drug dilution of seven 3-fold dilution steps covering a range from 90 to 0.123 μg/mL. After 96 h of incubation, the plates were inspected under an inverted microscope to assure growth of the controls and sterility. Then, the substrate CPRG/Nonidet (50 μL) was added to all wells. A color reaction developed within 2–6 h and could be read photometrically at 540 nm. The IC_50_ values were calculated from the sigmoidal inhibition curves with SoftMax Pro software. Benznidazole (Roche) was used as a positive reference.

#### 3.4.3. Activity against *Trypanosoma brucei rhodesiense*

The assays were performed according to the procedures described by [34]. For the extracts, working stock solutions of 180 µg/mL in serum containing culture medium according to Baltz *et al.* [35] were prepared. One-hundred microliters of the diluted extracts were pipetted in duplicate into the first row of a 96-well microtiter plate (Costar, USA). With the complete culture medium, three-fold serial dilutions were prepared. After the addition of *Trypanosoma brucei rhodesiense* bloodstream form trypanosomes from axenic culture, the concentrations of the extracts ranged from 90 µg/mL to 0.13 µg/mL and from 500 to 0.07 µg/mL for pure compounds. The total number of trypanosomes in each well was 2 × 10^3^/100 µL. The plate was then incubated for 72 h in a humidified atmosphere at 37 °C in 5% CO_2_. Ten microliters of resazurin solution (12.5 mg resazurin dissolved in 100 mL distilled water) were then added to each well, and incubation continued for a further 2–4 h. The plate was then read in a Spectramax Gemini XS microplate fluorometer (MolecularDevices Cooperation, Sunnyvale, CA, USA) using an excitation wavelength of 536 nm and an emission wavelength of 588 nm [34]. Fluorescence development was measured and expressed as the percentage of the control. Melarsoprol (Arsobal) was used as a positive reference.

### 3.5. Cytotoxicity against L6 Cells

Assays were performed in 96-well microtiter plates, each well containing 100 L of RPMI 1640 medium supplemented with 1% L-glutamine (200 mM) and 10% fetal bovine serum and 4 × 10^4^ L6 cells (rat skeletal myoblasts). Serial drug dilutions of seven 3-fold dilution steps covering a range from 90 to 0.123 μg/mL were prepared. After 72 h of incubation, the plates were inspected under an inverted microscope to assure growth of the controls and sterile conditions. Then, 10 μL of a resazurin solution (12.5 mg resazurin dissolved in 100 mL distilled water) was added to each well and the plates incubated for another 2 h. They were then read with a Spectramax Gemini XS microplate fluorometer at an excitation wavelength of 536 nm and an emission wavelength of 588 nm. The IC_50_ values were calculated from the sigmoidal inhibition curves with SoftMax Pro software. Podophyllotoxin (P4405, Sigma, Saint Louis, MO, USA) was used as a positive reference.

### 3.6. Calculation of IC_50_

To measure antiplasmodial activity, the concentration of extract at which the parasite growth (=[^3^H]hypoxanthine uptake) was inhibited by 50% (IC_50_) was calculated by linear interpolation between the two concentrations above and below 50% [36]. To assess leishmanicidal, antitrypanosomal and cytotoxic activity, we transferred data into the graphic SoftMax Pro program (Molecular Devices), which calculated IC_50_ values from the sigmoidal inhibition curve. The values given in Table 3 and Table 4 are the means of two independent assays.

## 4. Conclusions

Among the marine macrophytes species tested in the current study against *T. brucei rhodesiense*, only *B. bifurcata* showed strong activity. Bio-guided fractionation led to the identification of eleganolone, the main linear diterpene described in this species. Eleganolone showed only mild trypanocidal activity, but better antiplasmodial activity, suggesting trypanocidal activity could be due to minor compounds or to the synergy of several compounds separated during fractionation. Minor compounds of the active fractions will be further characterized by LC-HRMS-Cap NMR.
